# Different inflammatory responses are associated with *Ureaplasma parvum*-induced UTI and urolith formation

**DOI:** 10.1186/1471-2334-9-9

**Published:** 2009-01-26

**Authors:** Leticia Reyes, Mary Reinhard, Mary B Brown

**Affiliations:** 1 Department of Infectious Disease & Pathology, College of Veterinary Medicine, University of Florida, Gainesville, FL, USA

## Abstract

**Background:**

Epidemiologic studies show a strong association between *Ureaplasmas *and urogenital tract disease in humans. Since healthy humans can be colonized with *Ureaplasmas*, its role as a pathogen remains controversial. In order to begin to define the role of the host in disease, we developed a rodent model of urinary tract infection (UTI) using Fischer 344 (F344) rats. Animals were inoculated with sterile broth, 10^1^, 10^3^, 10^5^, 10^7^, or 10^9 ^log CFU of a rat-adapted strain of *Ureaplasma parvum*.

**Results:**

Infected animals exhibited two distinct profiles, asymptomatic UTI and UTI complicated with struvite urolithiasis. Inoculum dose of *U. parvum *affected the incidence of UTI, and 50% to 57% of animals inoculated with ≥ 10^7 ^CFU of *U. parvum *remained infected (p < 0.04). However, inoculum dose did not influence immune response to *U. parvum*. Asymptomatic UTI was characterized by a minimal immune response that was predominantly monocytic and lymphocytic, with limited lesions, and elevated urinary levels of IFN-γ, IL-18 and MCP-1 (P ≤ 0.02). UTI complicated with struvite formation was characterized by an exaggerated immune response that was mostly neutrophilic (P ≤ 0.0001), with lesions that showed extensive uroepithelial hyperplasia (P ≤ 0.0001), and a predominance of IL-1α, IL-1β, and GRO/KC in the urine (P ≤ 0.02). Animals with asymptomatic UTI also had a significantly high rate of kidney infection (P ≤ 0.0005).

**Conclusion:**

Complications associated with *U. parvum *infection are primarily dependent upon host-specific factors rather than *Ureaplasma *microbial load. The immune response in F344 rats is similar to that which occurs in humans with ureaplasmal associated disease. Therefore, this model of infection is a useful tool for elucidating *U. parvum*-host interactions that confer UTI and disease.

## Background

*Ureaplasma *species are among the most common isolates from the human urogenital tract [1,2]. Although ureaplasmas can be isolated from healthy individuals, epidemiologic studies have shown a strong association between Ureaplasmas and various diseases including non-gonococcal urethritis (NGU), bacterial vaginosis, infertility, prostatitis, epididymitis, urinary tract infection (UTI), nephrolithiasis, postpartum endometritis, chorioamnionitis, spontaneous abortion, premature birth, stillbirth and neonatal pneumonia [1-4]. Animal studies have demonstrated the ability of ureaplasmas to induce pneumonia, pyelonephritis, and struvite uroliths (urinary tract stones primarily composed of magnesium, ammonium, and phosphate) [5-10]. The most common host immune response to *Ureaplasma *during disease states involves elevated pro-inflammatory cytokines, most notably IL-1α, IL-1β, IL-6, IL-8, MCP-1 and TNF-α, accompanied by infiltration of neutrophils and macrophages at sites of infection [1,10-12]. However, little work has been done to characterize the immune response during uncomplicated infections. Therefore, the complex interactions between *Ureaplasma *and the host that lead to simple colonization versus inflammation and disease are largely unknown. In a recent study, we showed that the inbred rat strain Fischer 344 (F344) is susceptible to UTI induced by a rat adapted strain of *Ureaplasma parvum *isolated from the urine of a patient with recurrent UTI [13]. As part of that study, we found that 60% of infected F344 rats developed struvite uroliths, which were associated with an exaggerated inflammatory response that is similar to what has been reported in other disease states caused by *Ureaplasma *infection [1,5,6,11,12]. Interestingly, the other 40% of F344 rats developed uncomplicated UTI that was characterized by low concentrations of pro-inflammatory cytokines in urine as well as mild to moderate lesions in the lower urinary tract. Since F344 rats are an inbred strain, this particular infection model would be useful for identifying the host/*Ureaplasma *interactions that confer disease or asymptomatic infection without confounding variables that would be introduced by genetic variability.

In the study reported here, we examined the innate immune response to UTI induced with varying microbial concentrations of *U. parvum *in the F344 rat. By applying an integrated approach that combines histopathology with cytokine profiling, we were able to identify innate immune response profiles that were significantly different between an uncomplicated UTI and a UTI accompanied by struvite formation. Our findings provide insights into innate immune responses that are likely involved in the development of complicated disease with *Ureaplasma*.

## Methods

### *Ureaplasma *preparation and culture

A host-adapted strain of *U. parvum*, designated strain 257-48 was used for the entire study [13]. Fifty mls of *U. parvum *in logarithmic growth phase was aliquoted into 1 ml volumes and stored at -80°C. This stock was used for all experiments.

For infection studies, one ml of the working stock was grown in 45 ml of 10B broth for 12 to 16 hours at 37°C. The *Ureaplasma *culture was pelleted by centrifugation at 10,000 × g, at 4°C, for 50 minutes. Due to the delicate nature of *Ureaplasma*, the pellet was resuspended in 15 to 20 ml of fresh 10B broth instead of saline, to give a final concentration of 10^9 ^CFU per ml then serially diluted to produce various inocula that contained 10^7^, 10^5^, 10^3^, and 10^1 ^CFU per ml. The CFU of all inocula (including all serial dilutions) were confirmed by culture on A8 agar. For each infection experiment, at least two animals were included in each *U. parvum *dose group and experiments were replicated a minimum of 5 times.

Inocula and animal tissues were serially diluted 10-fold in 10B broth to 10^-10 ^and 10^-5^, respectively. For CFU determination, 20 μl from each sample and its corresponding dilutions were plated on A8 agar. Agar plates were incubated at 37°C in 5% CO_2_; broth cultures were incubated at 37°C in ambient air. Agar cultures were incubated for at least 5 days before colonies were counted to determine CFU.

### Animals

Specific pathogen free F344 virgin female rats were purchased from a commercial vendor (Charles River, Indianapolis, IN). All animals ranged in weight from 178–200 grams. Animal colonies were monitored and found free of the following pathogens: Sendai virus, H-1 virus, rat corona virus, sialodacroadenitis virus, reovirus type 3, Kilham rat virus, Hantaan virus, M. pulmonis, respiratory and enteric bacterial pathogens, endoparasites and ectoparasites. All animals were handled in accordance with procedures approved by the University of Florida Institutional Animal Care and Use Committee.

All animals were handled within a biosafety laminar flow hood. Rats were housed in Microisolator^® ^(Lab Products, Inc., Maywood, NJ) cages in the same room under the same temperature and light conditions. Control animals were always handled before infected and housed in separate microisolator cages in order to prevent contamination with *Ureaplasma*. All food, water, bedding, and caging were autoclaved before use.

Rats were anesthetized and inoculated with sterile broth or *U. parvum *inoculum into the bladder as previously described [13]. For each infection experiment, a minimum of two rats per inoculum dose were infected, so that each dose was represented in each experiment.

### Necropsy

Rats were necropsied at two weeks post-infection as previously described [13]. Prior to euthanasia, free catch urine was collected for cytokine analysis. The bladder was processed for histopathologic evaluation. Each kidney was transected sagittally so that a portion of the renal pelvis was present in each section. One half of each kidney was processed for histopathologic evaluation. The remaining halves of the right and left kidneys for an individual animal were combined, minced in sterile 10B broth, and the medium was aseptically removed and cultured for *U. parvum*.

### Stone analysis

Bladder calculi were submitted to a commercial laboratory (Louis C. Herring and Co., Orlando, FL) and analyzed by integrated crystallography.

### Histopathology

Bladder and kidney tissues were fixed in a paraformaldehyde-lysine-periodate [14] solution for 24 hours, then washed 3 times in sterile saline and transferred to 70% ethanol prior to processing. Tissues were processed routinely and stained with hematoxylin and eosin (H&E).

Bladder lesions were scored by a system developed in a previous study [13]. Epithelial changes in bladder tissues were scored as: 0 for none, 1 for minimal hyperplasia, ulceration or effacement of epithelium by inflammation; 2 for mild hyperplasia and rare dense inflammatory infiltrates, and 3 for the same changes noted in a score of 2 but accompanied with marked erosion and/or ulceration of the epithelial surface. Scoring for cell types that comprised the inflammatory infiltrate was: 1 for primarily mononuclear cells (lymphocytes, plasma cells and macrophages), 2 for mononuclear cells and neutrophils, and 3 for mononuclear cells, neutrophils and fibrous infiltrates. Kidney tissues were scored on the basis of total area affected, which was: 1 for less than 10%, 2 for 10 to 50%, and 3 for greater than 50%.

### Detection of urinary cytokines

Urine from control and infected rats was analyzed for the presence of cytokines with a multiplex antibody-immobilized bead immunoassay (Lincoplex KIT, Linco Research, Inc., St. Charles, MO). The manufacturer's protocol was followed for the simultaneous detection of the following cytokines and chemokines: GM-CSF, IL-1α, IL-1β, IL-6, IL-10, IL-12p70, IFN-γ, IL-18, GRO/KC (growth related oncogene/keratinocyte chemoattractant- the rat analog for human IL-8), and TNF-α within the same aliquot of urine. Briefly, a standard cocktail was serially diluted in order to develop a standard curve for each analyte that ranged from 3.2 to 2000 pg/ml. Urine samples were diluted in assay buffer to obtain a total volume of 60 μl per well, and run in duplicate as previously described [13].

### Data analysis

Data from multiple experiments were grouped together in order to make statistical analysis possible. Wherever possible, data were analyzed by one-way ANOVA when more than two groups were included in the analysis. Fisher's PLSD test was used when the ANOVA indicated a significant difference among group means. An unpaired student's T test was used for comparisons between two groups. Contingency table analysis was used for comparisons of groups involving nominal data (positive vs. negative). Cytokine pattern recognition analysis was performed using JMP Genomics 3.0 (SAS Institute, Cary, NC). Datasets were initially evaluated by distribution analysis and normalized prior to analysis by one-way ANOVA using row by row modeling and Fischer C correction for multiple comparisons. For all analyses a probability of P < 0.05 was considered significant.

## Results

### Impact of inoculum dose of *U. parvum *on colonization rates in the urogenital tract

*Ureaplasmas *were not isolated from any site from any control rat (data not shown). Neither uroliths nor crystals were detected at any site in animals inoculated with sterile 10B broth. There was no statistical difference in the log CFU of *U. parvum *isolated from culture positive animals among the various inoculum groups. The log CFU isolated from the bladder tissue of culture positive animals was 1.59 ± 1.4 (mean ± SD), and 1.48 ± 1.1 from kidney tissue. However, there was a statistical difference in the frequency of animals that remained infected 2 weeks post-inoculation (Table 1). Animals inoculated with 10^7 ^or higher CFU had the greatest frequency of bladder infections at time of necropsy (P ≤ 0.04).

**Table 1:** Colonization of rat urinary tract by *U. parvum*.

Number of rats positive (%) inoculated with log CFU *U. parvum*^a^						
	**10^1^**	**10^3^**	**10^5^**	**10^7^**	**10^9^**	**P value^b^**
**Bladder**	4/14 (29)	2/14 (14)	2/14 (14)	7/14 (50)	8/14 (57)	0.04
**Kidney**	1/14 (7)	2/14 (14)	3/14 (21)	1/14 (7)	6/14 (43)	NS
**Both Sites^c^**	1/14 (7)	1/14 (7)	1/14 (7)	2/14 (14)	4/14 (29)	NS

a) Data was collected at 2 weeks post-inoculation, and is a combination of 5 separate experiments.

b) Probability values were derived by G statistic, P values greater than 0.05 were considered not significant (NS).

c) Both sites refer to bladder and kidney.

### Clinical profiling of animals inoculated with *U. parvum*

Animals inoculated with *U. parvum *were divided into three groups (Negative, UTI, or Struvite) stratified on the basis of culture status and urolith status (Table 2). The Negative group consisted of animals found to be culture negative and urolith negative at time of necropsy. As expected, the frequency of animals that were culture negative after inoculation with *U*. *parvum *decreased as the log CFU of the inoculum increased. The UTI group represents animals that were culture positive in the bladder and/or kidney at time of necropsy but were found to be negative for uroliths. Eleven of 17 (64.7%) animals within the UTI group had kidney infections at time of necropsy, which was significantly greater (P ≤ 0.0005) than the number of renal infections within the urolith group [1 of 14 (7%) animals]. All animals within the Struvite group had bladder stones, which were composed of 89 to 95% magnesium ammonium phosphate (struvite), 1 to 5% calcium phosphate (carbonate apatite) and 3 to 10% protein and blood. No animal had macroscopic evidence of kidney stones at time of necropsy. Although not statistically significant (P ≤ 0.11), animals inoculated with 10^7 ^or greater CFU tended to have the highest frequency of struvite uroliths.

**Table 2:** Distribution of animals grouped within Negative, UTI, or Struvite profiles.

Inoculum dose of *U. parvum *(log CFU)						
**Profile^a^**	**10^1^**	**10^3^**	**10^5^**	**10^7^**	**10^9^**	**P value^b^**
**Negative**	10/14 (71)	11/14 (79)	9/14 (64)	6/14 (43)	3/14 (21)	0.012
**UTI**	2/14 (14)	2/14 (14)	4/14 (29)	3/14 (21)	6/14 (43)	NS
**Struvite**	2/14 (14)	1/14 (7)	1/14 (7)	5/14 (36)	5/14 (36)	NS

a) Profiles are based on culture status and urolith status of animals at time of necropsy. Negative group consists of animals found to be culture negative and urolith negative at time of necropsy. UTI group consists of animals that were culture positive in the bladder and/or kidney but urolith negative. Struvite group consists of animals found to have bladder uroliths regardless of *U. parvum *culture status at time of necropsy. Data is a combination of 5 separate experiments.

b) P values obtained by G statistic obtained by contingency analysis. NS = not significant.

The inoculum dose of *U. parvum *did not impact the distribution of animals that developed either UTI or struvite uroliths (Table 2). Further, there was no statistical difference between the log CFU cultured from bladder tissue among these two clinical groups. The log CFU (mean ± SD) of *U. parvum *isolated from the bladder of animals within the UTI group was 1.59 ± 2 and 1.53 ± 1 from the bladder of animals within the Struvite group.

### Histologic characterization of the inflammatory response to *U. parvum*

**Figure 1:**
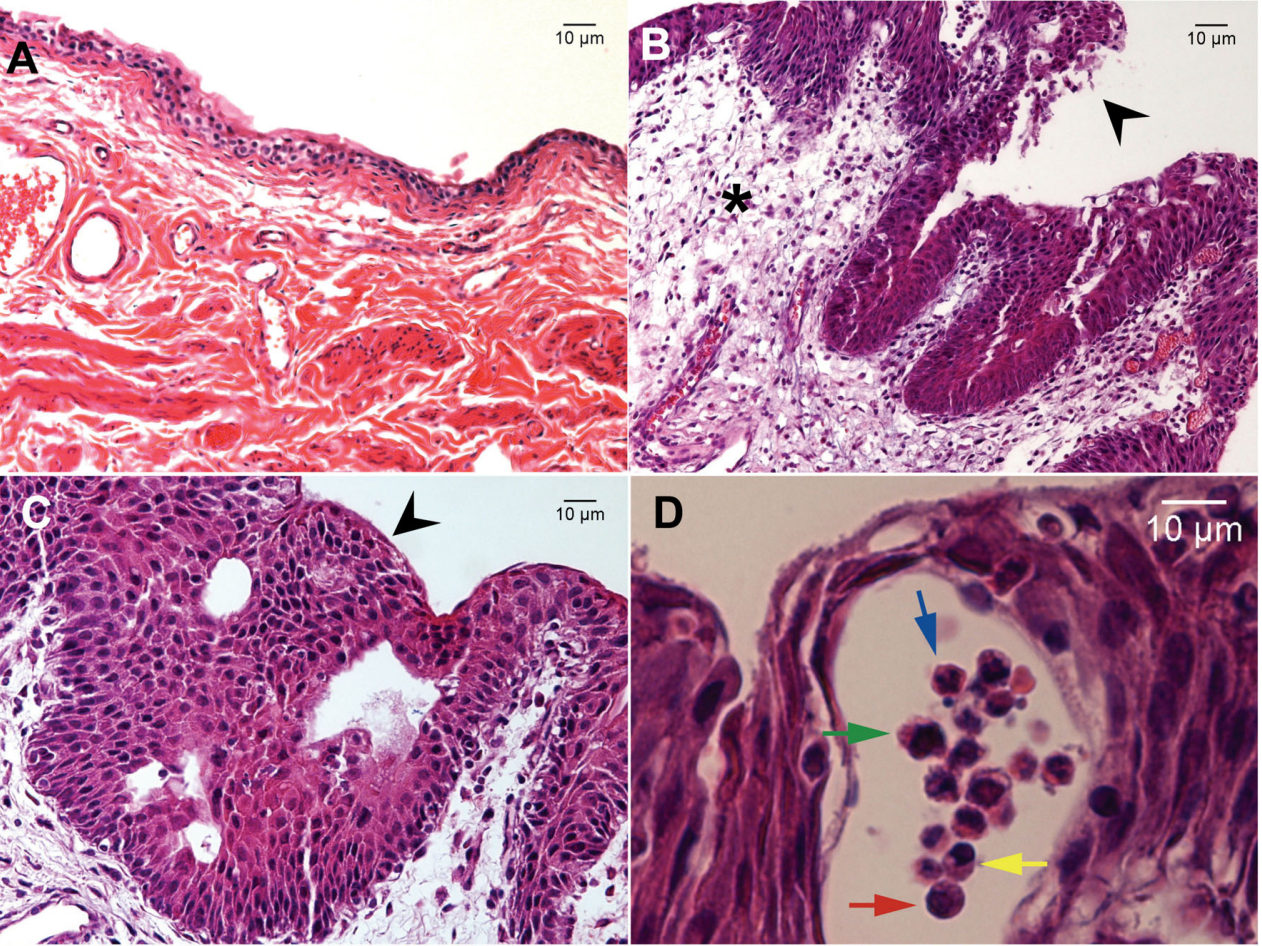
**Summary of the bladder lesions found in F344 rats experimentally infected with *U. parvum***. Panel A is a 200× magnification of bladder tissue from a Control rat. This sample represents a lesion score of 0 for degree of inflammation, degree of epithelial change and inflammatory cell type. Panels B, C, and D are tissue sections from animals inoculated with *U. parvum *that had a lesion score of 3 for degree of inflammation, degree of epithelial change and inflammatory cell type. Panel B is a 200× magnification of bladder tissue from an animal within the struvite group. The asterisk demarcates the extensive edema and fibrinous exudate infiltrating the submucosa. The black arrow points to uroepithelial effacement. Panel C is a 400× magnification of extensive uroepithelial hyperplasia. Panel D is a magnified inset of Panel B that highlights the array of white blood cells that comprised in the inflammatory cellular infiltrate in the tissues of infected animals. The blue arrow is pointing to a neutrophil. The green arrow is pointing to a tissue macrophage. The yellow arrow is pointing to a plasma cell. The red arrow is pointing to a lymphocyte.

**Figure 2:**
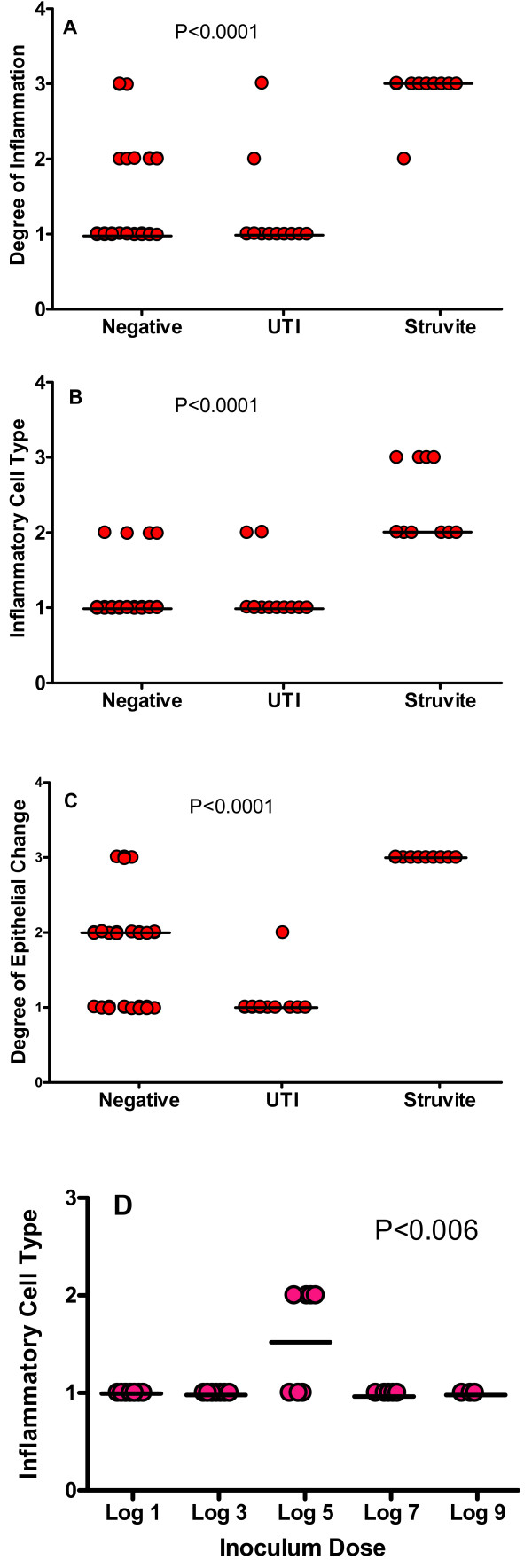
**Lesion scores of bladder tissue from F344 rats inoculated with *U. parvum***. Lesion score analysis was done prior to grouping each sample into a clinical profile (Panels A, B, and C) or inoculum dose groups (Panel D). Data is a summation of 5 separate experiments. In panels A, B, and C, nonparametric lesion scores were grouped according to the clinical profile and analyzed by Kruskall- Wallis test (Control, n = 6; Negative, n = 36; UTI, n = 11; Struvite, n = 10). Panel D are the results of an inflammatory cell type lesion score analysis performed on bladder tissues from animals in the Negative group. Scoring for cell types that comprised the inflammatory infiltrate was: 1 for primarily mononuclear cells (lymphocytes, plasma cells and macrophages), 2 for mononuclear cells and neutrophils, and 3 for mononuclear cells, neutrophils and fibrous infiltrates. Data is a summation of 5 separate experiments. Nonparametric lesion scores were grouped according to the inoculum dose of *U. parvum*. Raw lesion scores were analyzed by Kruskall- Wallis test (n = 10 for log 1 CFU, n = 11 for log 3 CFU, n = 8 for log 5 CFU, n = 5 for log 7 CFU, and n = 3 for log 9 CFU). Values in all graphs represent raw lesion scores for each biological replicate. Horizontal bars demarcate the median value for each clinical profile group.

The extent and severity of bladder lesions as well as the types of inflammatory cells present differed among groups. There were no detectable lesions in bladder tissue from control rats (see Figure 1, panel A). In animals inoculated with *U. parvum*, bladder lesions associated with inflammation were highly variable. When present, lesions consisted of infiltrates of lympho-plasma cells, macrophages, and neutrophils that were primarily located within the epithelial layer (Figure 1, panel B and D). Mast cells could be found within the submucosa regardless of clinical profile (data not shown). Uroepithelial changes ranged from spongiosis of epithelial cells with some necrosis, exfoliation of uroepithelium, or hyperplasia (see Figure 1, panels B, C, and D). Animals with struvite uroliths had the most extensive lesions and the highest lesion scores (Figure 2). In these animals, inflammation extended into the submucosa and muscularis layers, and was occasionally accompanied by venous congestion and edema with a fibrinous reaction (Figure 1, panel B). Neutrophilic infiltrates were present in all of the animals within the Struvite group. In contrast, infiltrates in animals within the Negative and UTI groups were predominantly lymphocytes, plasma cells and macrophages (Figure 2, panel B). Animals with uroliths also had the most extensive uroepithelial hyperplasia as shown in Figure 1, panel C. Animals within the Negative and UTI groups had the mildest inflammatory changes (Figure 2). However, somewhat surprisingly, more animals within the Negative group had a higher degree of both inflammation and epithelial change than did animals within the UTI group. Most of the animals in the UTI group exhibited exfoliation of uroepithelium with some spongiosis.

Both the inflammatory cell type score and inoculating dose of *U. parvum *influenced the lesion scores within the Negative group. There was no difference in the lesions scores pertaining to degree of inflammation and degree of epithelial change among the inoculating dose groups (data not shown). However, there was a significant difference (P ≤ 0.006) in the inflammatory cell type score and inoculating dose of *U. parvum *(Figure 2, panel D). A significant number of animals that received log 5 CFU had a mixed inflammatory cell infiltrate that included neutrophils as well as mononuclear cells (lymphocytes, plasma cells, and macrophages).

**Figure 3:**
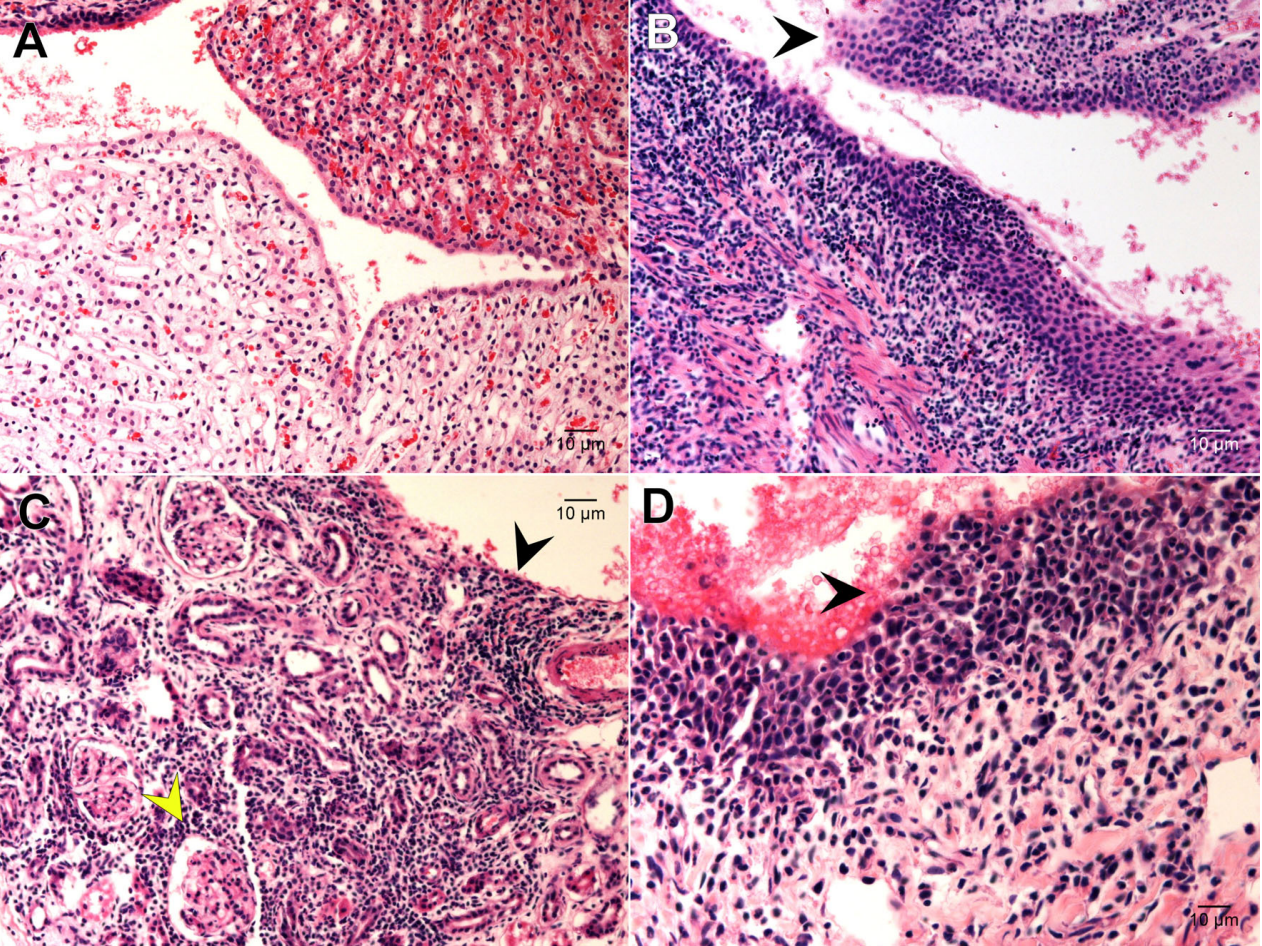
**Summary of the kidney lesions found in F344 rats experimentally infected with *U. parvum***. Panel A is a 200× magnification of kidney tissue from a Control rat and demonstrates the lack of inflammatory lesions that are characteristic in animals inoculated with *U. parvum*. Panels B, C, and D are tissue sections from animals inoculated with *U. parvum *that had a lesion score of 4 for total area affected. Panel B is a 400× magnification demonstrating the inflammatory infiltrate extending from the renal pelvic space into the interstitium with uroepithelium largely intact. The black arrow is pointing to uroepithelial hyperplasia. Panel C is a 400× magnification of renal tubules. The black arrow is pointing to the extensive inflammatory infiltrate throughout the renal tubular interstitium. The yellow arrow is pointing to a glomerulus. Panel D is a 600× magnification of renal uroepithelium at the edge of the pelvic space. The black arrow is pointing to extensive hemorrhage and disruption of the uroepithelial barrier by a fibrinous inflammatory infiltrate.

Kidney tissue from F344 rats were also evaluated for the presence of inflammation. There were no significant lesions present in the collecting ducts and renal pelvis of control rats (Fig 3, panel A). Histopathologic findings in kidney tissue from rats inoculated with *U. parvum *ranged from minimal changes to varying degrees of inflammatory infiltration that consisted of lymphocytes, plasma cells, macrophages and neutrophils. In some animals, lesions were characterized by scant multifocal areas of predominantly mononuclear cells that were present in the subepithelial region of the renal pelvis (Figure 3, panel B). In these animals, the uroepithelial lining the pelvic space was hyperplastic. Animals with the most severe kidney lesions had extensive erosion of the uroepithelium, as shown in Figure 3, panel D. In these animals, erosion of the uroepithelial barrier was accompanied by hemorrhage and infiltration with inflammatory cells (predominantly neutrophilic) that spanned the pelvic luminal space, through the epithelial layer and into the sub-epithelial region of the pelvis (Figure 3, panel D). Other animals, had inflammatory infiltration of the renal interstitium as shown in Figure 3, panel C

Scoring of kidney tissues on the basis of total area affected did not reveal any patterns that could be correlated to local/active infection or clinical profile. Specifically, there was no correlation between the total area affected score and *U. parvum *culture status (data not shown). There also was no correlation between the total area affected score and clinical profile (Negative, UTI, or Struvite), additional file 1.

### Urine cytokine analysis of clinical profiles

The relationships between specific cytokines and bladder lesion scores among animals inoculated with sterile 10B broth or *U. parvum *were examined by Spearman Correlation analysis. There were no correlations between urine cytokine levels and degree of inflammation score among animals inoculated with sterile 10B broth (data not shown). However, there was a significant correlation (summarized in Table 3) between the degree of inflammation score and urine concentrations of GRO/KC, IL-1α, IL-1β, IL-10 and TNF-α. There was also a significant correlation between degree of epithelial change and urine concentrations of GRO/KC and IL-1β (Table 4). MCP-1 levels negatively correlated with degree of epithelial change (Table 4).

**Table 3:** Urine chemokines/cytokines found to have a significant correlation to Degree of Inflammation^a^

Cytokine	Rho^b^	Tied Z value	Tied P value
GRO/KC	0.462	3.55	0.0004
IL-1α	0.382	2.94	0.003
IL-1β	0.652	5.01	0.0001
IL-10	0.351	2.70	0.007
TNF-α	0.359	2.76	0.006

a) Data was a combination of 5 separate experiments (n = 58).

b) Spearmann correlation analysis was performed on cytokine data from animals inoculated with *U. parvum *regardless of their clinical profile grouping. Listed Rho values have been corrected for tied lesion scores.

**Table 4:** Urine chemokines/cytokines found to have a significant correlation to Degree of Epithelial Change^a^

Cytokine	Rho^b^	Tied Z value	Tied P value
GRO/KC	0.370	2.79	0.0053
IL-1β	0.407	3.08	0.0021
MCP-1	**-0.270**	**-2.04**	0.042

a) Data was a combination of 5 separate experiments (n = 58).

b) Spearmann correlation analysis was performed on cytokine data from animals inoculated with *U. parvum *regardless of their clinical profile grouping. Listed Rho values have been corrected for tied lesion scores.

**Figure 4:**
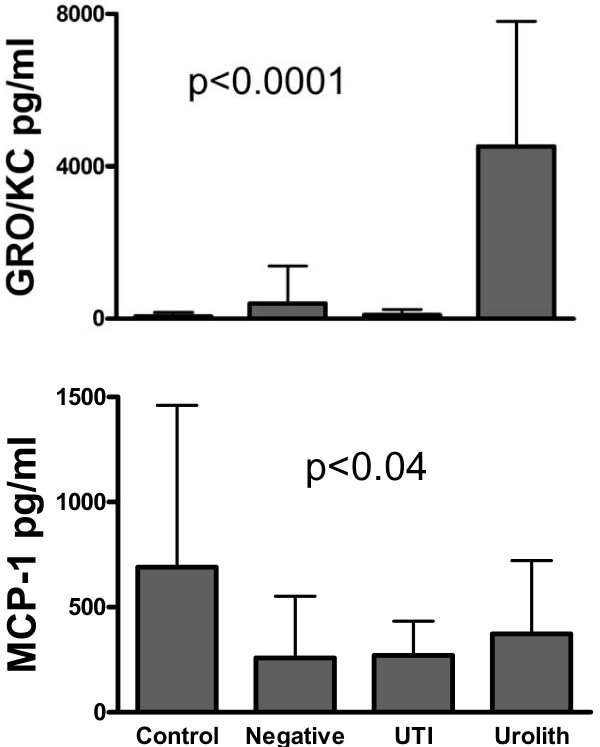
**Urine chemokines detected in F344 rats inoculated with sterile 10B or *U. parvum***. Data represent the mean ± SD of a combination of 5 separate experiments. Urine chemokine concentrations were grouped according to clinical profile, control (n = 6), negative (n = 36), UTI (n = 16), and Struvite (n = 13). P values within each graph were obtained by one-way ANOVA. Fisher's PLSD test revealed that GRO/KC concentrations in the struvite group were significantly greater than the control, negative and UTI groups. Fisher's PLSD test revealed that MCP-1 concentrations in the control group was significantly greater than the negative and control groups.

**Figure 5:**
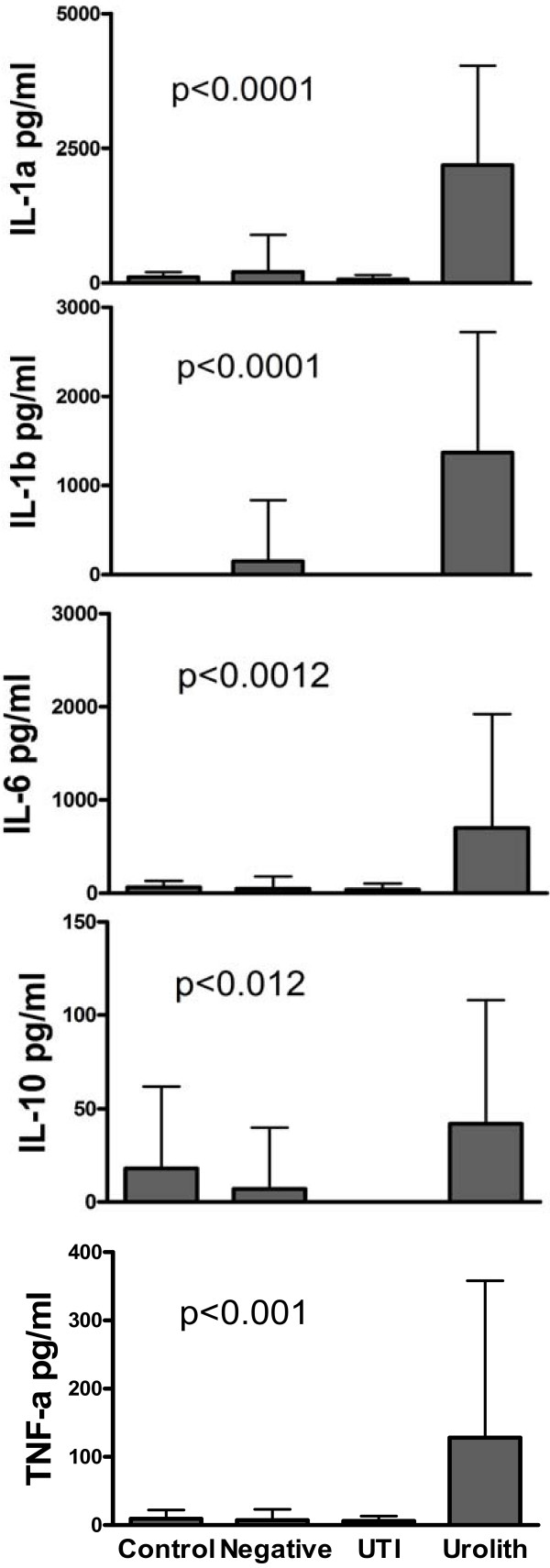
**Urine cytokines detected in F344 rats inoculated with sterile 10B or *U. parvum***. Data represent the mean ± SD of a combination of 5 separate experiments. Urine cytokine concentrations were grouped according to clinical profile, control (n = 6), negative (n = 36), UTI (n = 16), and Struvite (n = 13). P values within each graph were obtained by one-way ANOVA. Fisher's PLSD test revealed that IL-1α, IL-1β, IL-6, and TNF-α concentrations in the struvite group were significantly greater than control, negative, and UTI groups. Fisher's PLSD test revealed IL-10 concentrations in the struvite group were significantly greater than negative and UTI groups.

The cytokine profile in urine differed among groups. Absolute concentrations of each individual cytokine in urine were compared among clinical profiles (Control, Negative, UTI and Struvite). Control animals had significantly higher levels of MCP-1 than animals in either the Negative or UTI groups (Figure 4). Animals in the Struvite group had the highest levels of GRO/KC (Figure 4), IL-1α, IL-1β, IL-6, IL-10 and TNF-α (Figure 5) than did animals in all other groups. There was no statistical difference in the absolute amounts of urine GM-CSF, IFN-γ, IL-2, IL-4, IL-12, and IL-18 among groups (data not shown).

**Figure 6:**
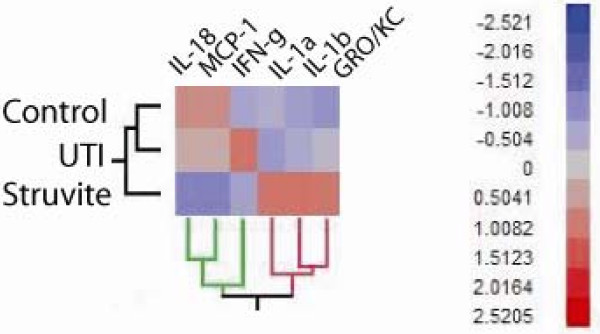
**Global profiling of urine cytokines detected in control and *U. parvum *infected F344 rats**. The clustered heat map represents the standardized LS means for each cytokine that had a significantly different pattern of expression among clinical groups (P ≤ 0.02). Values were obtained by one-way ANOVA using a row by row modeling with Fischer C correction for multiple comparisons. Two main cytokine cluster patterns were identified in the analysis and are demarcated by the green and red cluster tree. The number of biological replicates were n = 6 for control, n = 16 for UTI group, n = 13 for the Struvite group.

In order to identify distinctive chemokine/cytokine patterns between clinical profiles associated with active infection, samples from animals within the Negative group were excluded from this analysis. Each urine cytokine multiplex from each animal was normalized prior to analysis by one-way ANOVA using row by row modeling and Fischer C correction for multiple comparisons. Figure 6 is a clustered heat map illustrating two cytokine profile clusters that significantly differed between Control, UTI and Struvite groups (P ≤ 0.02). Both Control and UTI groups showed a significant emphasis in IL-18 and MCP-1, whereas the Struvite group showed a significant emphasis in IL-1α, IL-1β, and GRO/KC. Only the UTI group showed a significant emphasis in IFN-γ.

### The impact of inoculum dose on the urine cytokine profiles of animals in the Negative group

**Figure 7:**
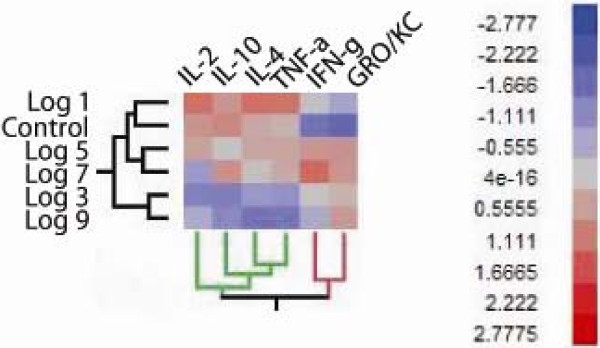
**Profiling the inflammatory response to different doses of *U. parvum *in culture negative F344 rats**. Panel A is a clustered heat map representing the standardized LS means for each cytokine with a significantly different pattern of expression among infection dose groups (P 0.05). Values were obtained by one-way ANOVA using a row by row modeling with Fischer C correction for multiple comparisons. Two main cytokine cluster patterns were identified in the analysis and are demarcated by the green and red cluster tree. The number of biological replicates were n = 6 for control, n = 10 for log 1 CFU, n = 11 for log 3 CFU, n = 8 for log 5 CFU, n = 5 for log 7 CFU, and n = 3 for log 9 CFU. The red arrow is highlighting the pattern of cytokines present in the urine of culture negative rats that were inoculated with log 5 CFU. Animals within this dose group were the only animals to exhibit an obvious inflammatory cell infiltrate comprising a mixture of mononuclear cells with neutrophils (P 0.006, Figure 2, Panel D).

Urine cytokine data from control and culture negative animals was normalized prior to statistical analysis and analyzed as described above. There was a significant difference in the overall pattern of IL-2, IL-4, IL-10, TNF-α, IFN-γ, and GRO/KC in the urine of culture negative animals that received different inoculating doses of *U. parvum *(P ≤ 0.05). Figure 7 is a clustered heat map illustrating two distinct cytokine profile clusters (green and red cluster trees) among these animals. With the exception of log 5 and log 7 inoculating groups, there was an inverse relationship in the pattern of expression between the two cytokine clusters.

## Discussion

Ureaplasmas are an underappreciated pathogen of the urogenital tract. Despite strong epidemiological evidence and even experimental infections in humans that fulfilled Koch's postulates [15], the etiologic role of *Ureaplasmas *is confounded by the isolation of the microbe from the lower urogenital tract of normal, asymptomatic individuals. In addition, the severity of disease for most mycoplasmal infections depends on the host immune response. Therefore, experimental infections in genetically defined animal models will be critical to unraveling the key interactions in the host/parasite relationship that contribute to disease severity. By using a combined approach involving histopathology and cytokine profiling, we were able to further characterize the immune response associated with asymptomatic UTI and UTI complicated with struvite formation.

The F344 rat strain is highly susceptible to development of complicated UTI following experimental inoculation with *U. parvum *[13]. In this study, we showed that varying the inoculum dose of *U. parvum *significantly affected the frequency of animals that remained colonized two weeks after inoculation. Therefore, the initial microbial load is important in establishing infection. However, once infected, the proportion of animals that developed complicated UTI in response to varying inocula of *U. parvum *did not show a definitive dose response effect. More importantly, the immune response to infection in this group of animals with complicated UTI was consistent, regardless of initial inoculating dose. For example, the cytokine profile and urinary tract pathology of a struvite positive animal that was inoculated with 10^1 ^CFU was indistinguishable from a struvite positive animal that was inoculated with 10^9 ^CFU. This suggests that the initial microbial load of *U. parvum *is not a critical factor in the development of complicated UTI in this rat strain. Further, it supports the concept that, once infection is established, the host inflammatory response is a key determinant of lesion severity in the urinary tract.

As previously reported [13], animals with asymptomatic UTI had significantly less pro-inflammatory urine cytokines and tissue damage when compared to rats with struvites. By profiling the entire cytokine milieu, we were able to identify a significant predominance of cytokines such as IFN-γ, IL-18, and MCP-1 in the UTI group that work synergistically to regulate monocyte/macrophage activation [16-18]. An intriguing finding was the significant emphasis of IFN-γ in the urine of animals with asymptomatic UTI, since this cytokine is a potent priming agent for macrophages [19]. This cytokine profile also coincides with the cellular immune response in these animals that consisted of macrophages, lymphocytes, and plasma cells, which resembles a profile that may be seen during the healing or resolution phase of infection. We cannot rule out that these animals could be displaying a pre-resolution phase to infection, but there are indicators suggesting that these animals have compromised immune defense. For example, the immune profile of these animals was obtained while they were actively colonized with *U. parvum*, and 65% exhibited an ascending infection into the kidneys. Further, the microbial load of *U. parvum *in animals with asymptomatic UTI was equivalent to animals in the Struvite group. Another intriguing feature in animals with asymptomatic UTI was the overall lack of uroepithelial proliferation that was present in varying degrees in the Negative group as well as the Struvite group. A primary defense mechanism of uroepithelium exposed to bacteria involves desquamation, necrosis or apoptosis followed by proliferation [20]. Therefore, the overall lack of this response in animals with asymptomatic UTI also implies that uroepithelial defense mechanisms may be perturbed by *U. parvum*.

F344 rats with struvite uroliths had a similar clinical profile to what we have previously described [13]. Specifically, these animals had the greatest concentration of pro-inflammatory cytokines in their urine (GRO/KC – the rat analog of human IL-8, IL-1α, IL-1β, IL-6 and TNF-α) and the most extensive inflammatory changes in bladder tissue. Since IL-1β is a known inducer of IL-8 and GRO chemokines in human and murine epithelial cells [21-23], it is not an unexpected finding that these cytokines are closely linked in their expression. Cytokine pattern analysis showed this cytokine cluster is unique to animals with struvites. Moreover, there is a significant positive correlation between IL-1β, GRO/KC and the degree of histopathologic change, which suggests that IL-1β and GRO/KC are critical elements in a pro-inflammatory loop that leads to chronic active inflammation, epithelial hyperplasia and struvite formation as seen in struvite positive F344 rats. Most of the animals within the struvite group had uroepithelial hyperplasia or erosion with hemorrhage and inflammation within the kidneys, yet none of these animals had uroliths in the renal pelvis that could account for these lesions. Therefore, although mechanical irritation by the urolith itself may partially contribute to epithelial erosion or hyperplasia in the bladder, it cannot entirely account for the lesions that were present in the urinary tract of these animals.

The immune response of animals within the negative group was highly variable and most likely comprises a mixed population of rats, including animals that never became colonized as well as animals that cleared the infection at various time points post-inoculation. Therefore, interpretation of data from this group of animals is difficult and is done with caution. In spite of this limitation, profiling urine cytokine data and bladder lesion scores by inoculum dose was informative. The threshold dose for successful colonization appears to be between log 5 and log 7 CFU, since 64% and 43% of rats respectively were culture negative 2 weeks post-inoculation. The animals within the log 5 and log 7 CFU inoculation groups also had the greatest flux in both pro-inflammatory (TNF-α, IFN-γ and GRO/KC) and anti-inflammatory (IL-4, and IL-10) cytokines. Interestingly, pattern analysis of urine cytokine data showed two distinct clusters. The first cluster identified in the negative group included IL-2, IL-4, IL-10, and TNF-α; these cytokines were notable as they were not part of the cytokine cluster groupings of *U. parvum *infected animals. Therefore, these cytokines may be critical in directing a more efficient immune response that leads to bacterial clearance with minimal tissue damage. The second cytokine cluster in the negative group included IFN-γ and GRO/KC, which are significant cytokines in the UTI and Struvite groups, respectively. Except for animals that were inoculated with log 9 CFU of *U. parvum*, the expression pattern of IFN-γ and GRO/KC was not inversely related as they were in culture positive animals. This may be reflecting a more balanced immune response than what is seen in *U. parvum *positive animals, and we suggest that this balanced response may be critical to resolution of infection and prevention of severe disease.

The variable clinical outcome to experimental inoculation with *U. parvum *in the F344 rat is an interesting phenomenon since this is an inbred strain. In this study, both genetic and environmental influences on disease were minimized to the extent possible. All of the animals in this study originated from the same colony. Further, rats were housed under the same barrier maintained conditions in order to minimize environmental variability. Despite our efforts, it was common to find that a rat that developed asymptomatic UTI had co-habited the same cage with a rat that developed struvites or was culture negative at time of necropsy. Therefore, external environment could not account for the varying clinical outcome in our study. However, our experimental inoculation procedure may be a critical source of variability. Although attempts were made to reduce mechanical trauma caused by catherization, it is possible that the trauma may have been sufficient to shift the immune response towards a pro-inflammatory profile in a subset of animals. Once this occurred, the pro-inflammatory cycle progressed until infection was resolved (Negative group) or the study was terminated (Struvite group). Another possible explanation for our findings may involve the actual placement of ureaplasmas within the urinary tract at time of inoculation. For example, if the catheter disrupted the uroepithelial barrier so that a sufficient number of ureaplasmas were deposited into the submucosa instead of the mucosal surface, this could elicit a different inflammatory response cascade than what would normally occur if the microorganisms were only present on the mucosal surface of the bladder. The results of this study were similar to what we have previously reported, thus showing the consistency and reproducibility of this model of infection. Moreover, the clinical outcome to ureaplasmal infection in the F344 rat is similar to what occurs in humans. The complex interactions between most mucosal pathogens and the host that lead to uncomplicated colonization versus inflammation and disease are largely unknown. Therefore, this model may be particularly useful for identifying the molecular events that confer asymptomatic infection, complicated infection as well as resolution of infection with an opportunistic pathogen of the urogenital tract.

## Conclusion

The complex interactions between *Ureaplasma *and the host that lead to uncomplicated colonization versus inflammation and disease are largely unknown. We characterized the F344 rat immune response in the urinary tract to varying inoculum concentrations of *U. parvum*. Establishment of UTI was influenced by microbial load, but the host immune response was independent of microbial load. Two distinct innate immune profiles were identified with two different clinical outcomes: asymptomatic UTI and complicated UTI with struvite formation. Asymptomatic UTI was characterized by a minimal immune response that was predominantly monocytic and lymphocytic and was accompanied by a significantly high rate of kidney infection. UTI complicated with struvite formation was characterized by an exaggerated immune response that was predominantly neutrophilic and was accompanied by uroepithelial hyperplasia and extensive tissue damage.

## Competing interests

The authors declare that they have no competing interests.

## Authors' contributions

LR designed and executed animal infection studies, data analysis and manuscript preparation. MR performed histopathologic evaluation of tissues and developed the lesion scoring system implemented in this study. MBB participated in the design and coordination of the study, and helped draft the manuscript. All authors read and approved the final manuscript.

## Pre-publication history

The pre-publication history for this paper can be accessed here:

http://www.biomedcentral.com/1471-2334/9/9/prepub

## Supplementary Material

Additional file 1 **Distribution of kidney lesion scores in F344 rats inoculated with varying doses of *U. parvum***. Lesion score analysis of kidney tissue was based on total area affected. The data provided represents the raw lesion scores for each biological replicate. Horizontal bars demarcate the median value for each clinical profile group. Lesion scoring of each sample was performed without prior knowledge of the clinical profile. Data is a summation of 5 separate experiments. Nonparametric lesion scores were grouped according to the clinical profile and analyzed by Kruskall-Wallis test (Control, n = 6; Negative, n = 36; UTI, n = 11; Struvite, n = 10). Click here for file
