# Supplementary material for: Different inflammatory responses are associated with Ureaplasma parvum-induced UTI and urolith formation
Source: BMC Infect Dis. 2009 Jan 26;9:9. doi: 10.1186/1471-2334-9-9 (PMC2656517; doi:10.1186/1471-2334-9-9)
Supplement: Supplementary file 1 [file 1471-2334-9-9-S1.rtf]

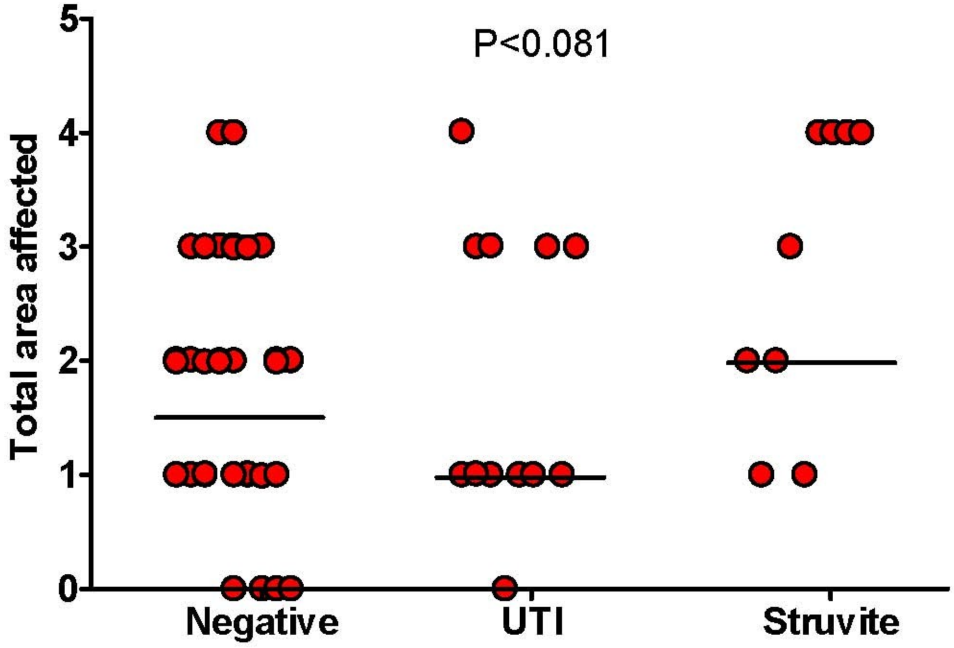

P<0.081
Total area affected
5
4
3
2
1
0
Negative
UTI
Struvite
